# Supplementary material for: Arginine Methylation Regulates Ribosome CAR Function
Source: Int J Mol Sci. 2021 Jan 29;22(3):1335. doi: 10.3390/ijms22031335 (PMC7866298; doi:10.3390/ijms22031335)
Supplement: Supplementary file 1 [file ijms-22-01335-s001.zip › ijms-1065883-supplementary/supplementary_materials_revised_1_25.pdf]

Supporting Materials

Arginine Methylation Regulates Ribosome CAR Function

Kristen Scopino, Carol Dalgarno, Clara Nachmanoff, Daniel Krizanc, Kelly M. Thayer and Michael P. Weir*

Department of Biology, Department of Mathematics and Computer Science, Department of Chemistry, College of Integrative Sciences, Wesleyan University, Middletown, CT 06459
* Correspondence: mweir@wesleyan.edu

1. Supplementary Materials

1.1 Supplementary Figures

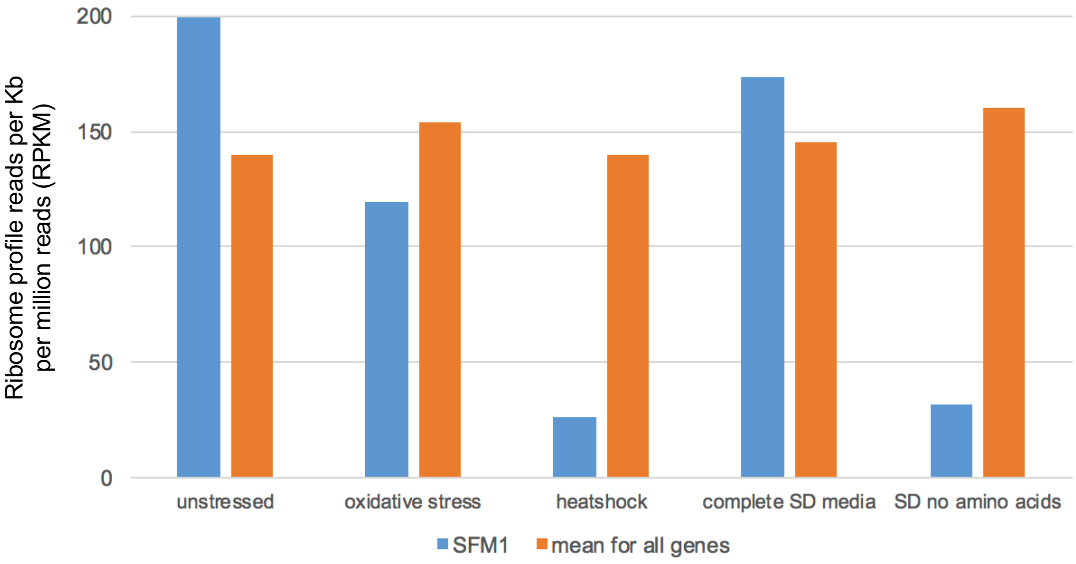

Figure S1. *SFM1* expression is depressed in stress conditions.
Analysis of ribosome profile data of Gerashchenko and Gladyshev [21] revealed that Sfm1, the methyl transferase that methylates Rps3 R146, has reduced expression in stress conditions.

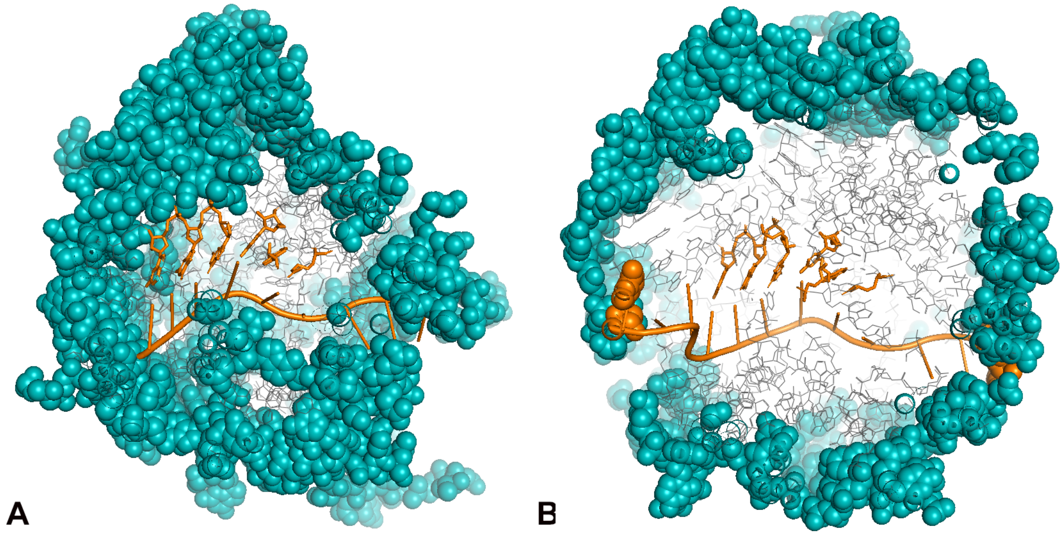

Figure S2. Design of N1 and N2 subsystem neighborhoods.
Subsystem N1 was used previously [12] in analysis of the CAR interface. Subsystem N2, used in this study, is more centered over the A site and CAR interface, and exhibits similar behavior to N1. Na^+ ions were used to achieve electro-neutrality in N1, and K^+ ions were used for N2.

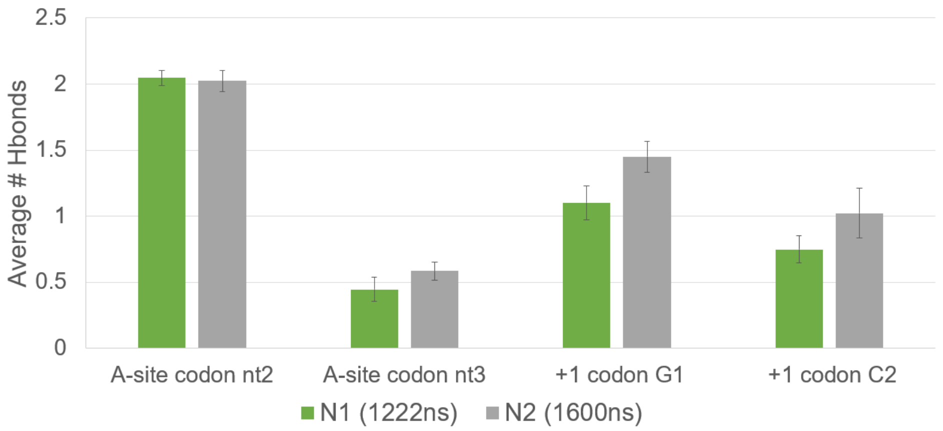

Figure S3. CAR H-bonding in neighborhoods N1 and N2.
Subsystems N1 and N2 were compared using +1 codon GCU. N2 had slightly greater H-bonding between CAR and G1 or C2 of the +1 codon, although the differences were not significant.

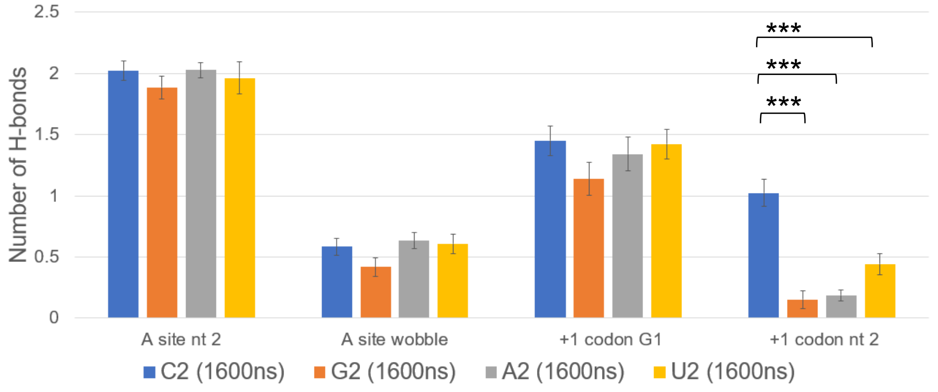

Figure S4. C2 substitutions in the +1 codon reduce CAR H-bonding.
As observed previously with subsystem N1 [12], the N2 subsystem also revealed that H-bonding is reduced when the second nucleotide of the +1 codon (C2) is replaced with G2, A2 or U2 ($p < 0.001$ ***), suggesting that N1 and N2 behaviors are similar.

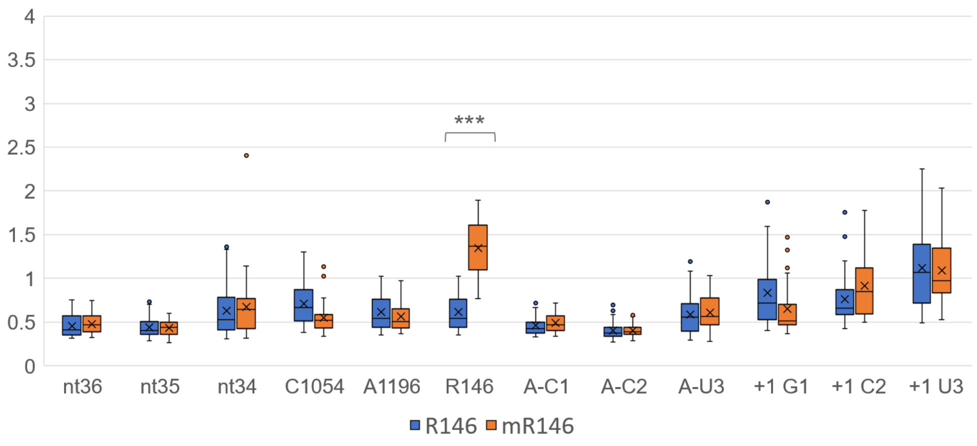

Figure S5. mR146 has elevated RMSF
Like the N2 subsystem (Figure 2), the N1 subsystem showed that mR146 has elevated RMSF compared to R146 for core base heavy atoms of nucleotides and guanidinium group heavy atoms ($p < 0.001$ ***).

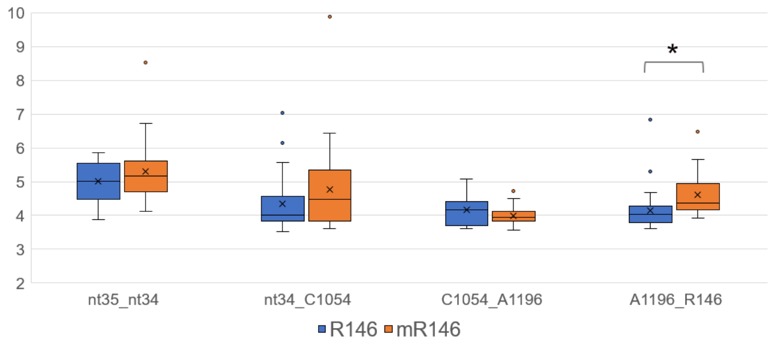

Figure S6. mR146 has reduced stacking.
Like the N2 subsystem (Figure 3A), the N1 subsystem showed that mR146 has reduced pi stacking compared to R146 ($p < 0.05$ *), indicated by increased distance between centers of mass of the A1196 base and the guanidinium group.

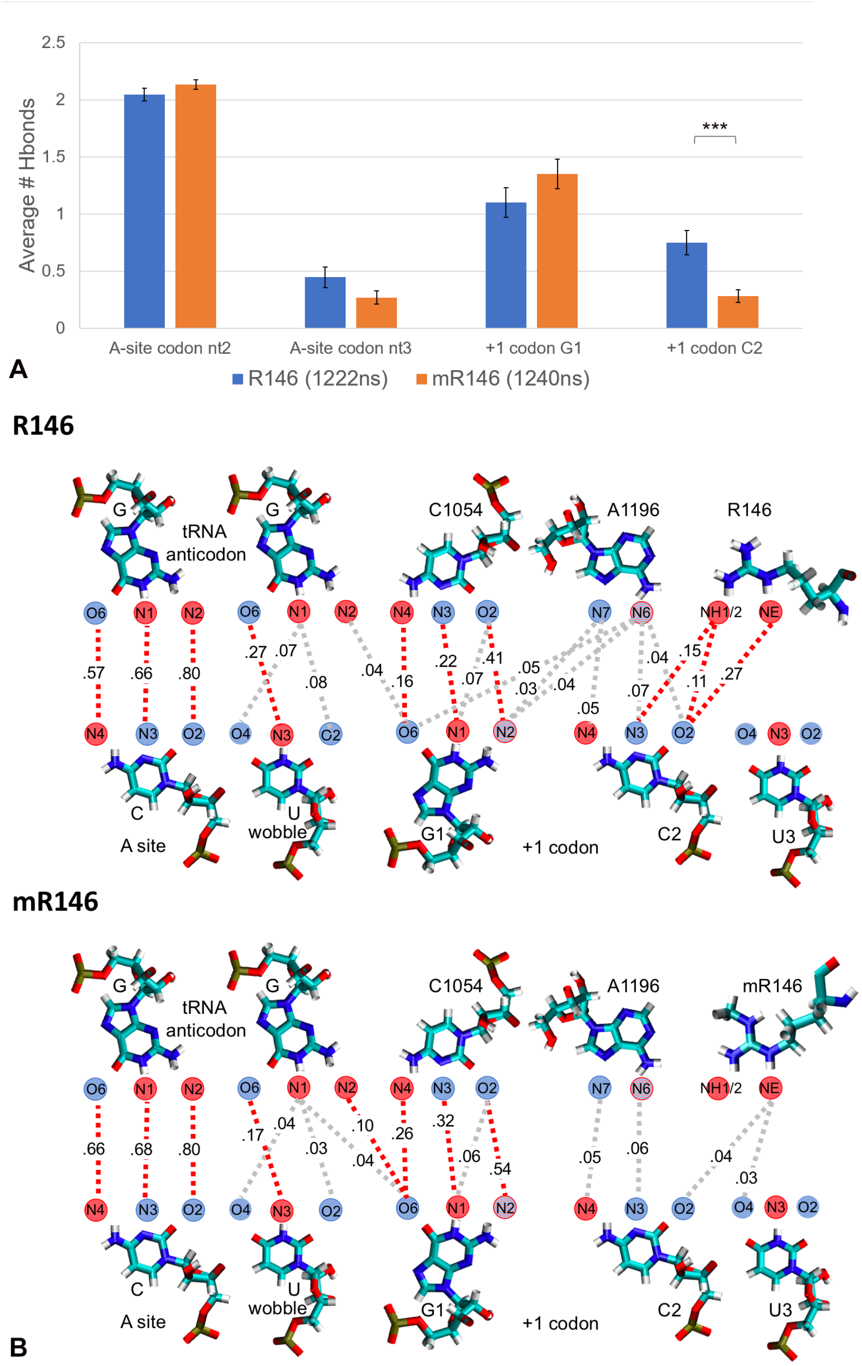

Figure S7. mR146 has reduced CAR/+1 codon H-bonding.
(A) Like the N2 subsystem (Figure 4), the N1 subsystem showed that mR146 had reduced H-bonding between the CAR interface and the +1 codon. +1 codon C2 had significantly reduced H-bonding ($p < 0.001$ ***). (B) Details of H-bonding interactions.

A. R146 60 ns

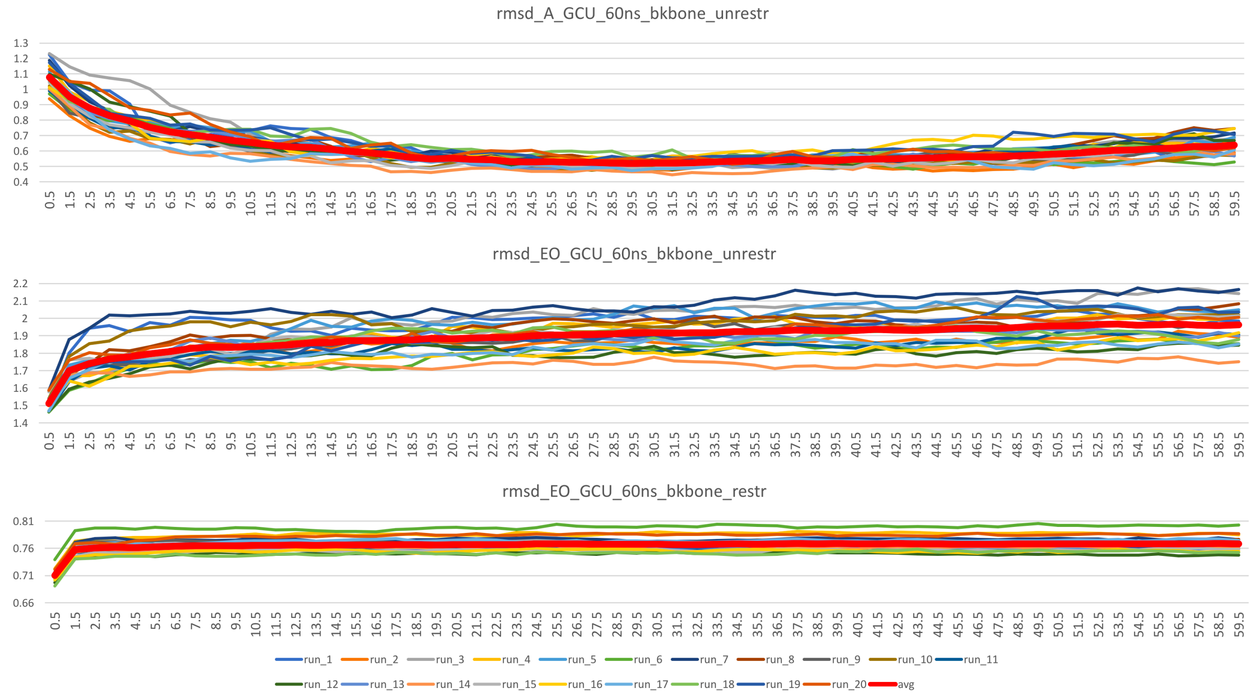

B. R146 100 ns

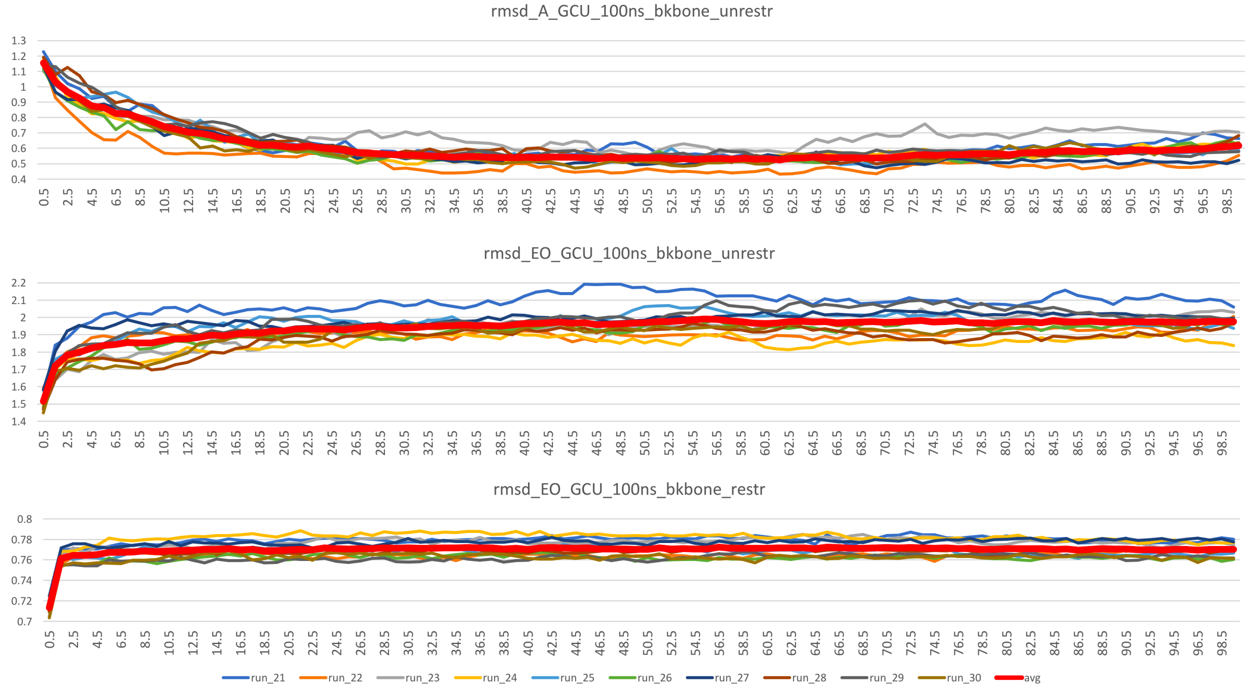

C. mR146 60 ns

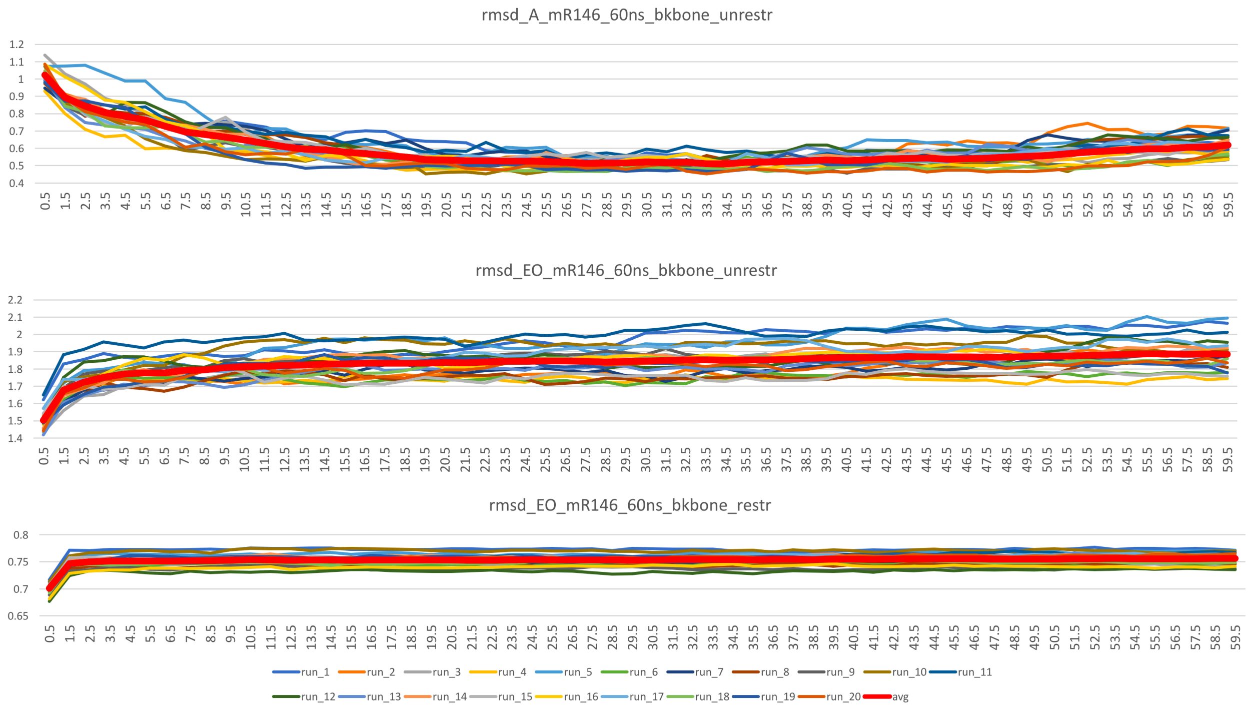

D. mR146 100 ns

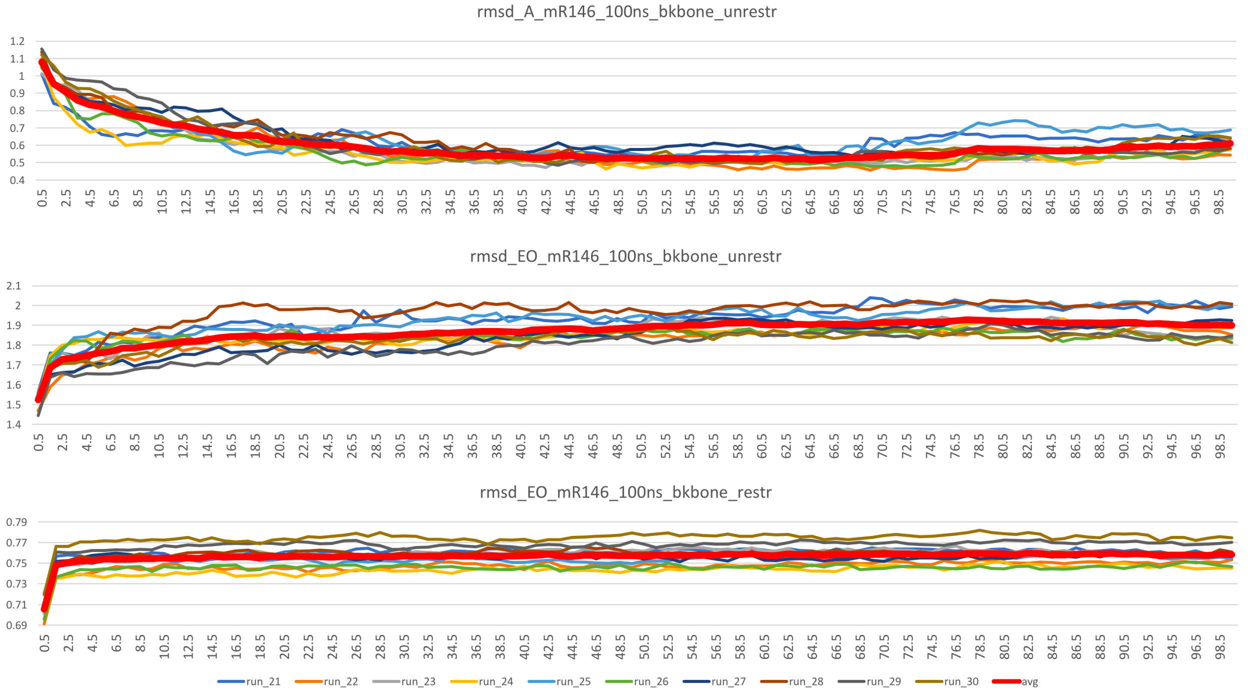

Figure S8. RMSD profiles for R146 and mR146 in neighborhood N2.
RMSD profiles were computed using as reference the average structure across the full trajectory (RMSD_A) or the structure at the end of equilibration (RMSD_EO). R146 and mR146 subsystems were analyzed using 60- and 100-ns trajectories. The trajectories stabilized by 20 ns and the first 20 ns of trajectories were not used for further analysis.

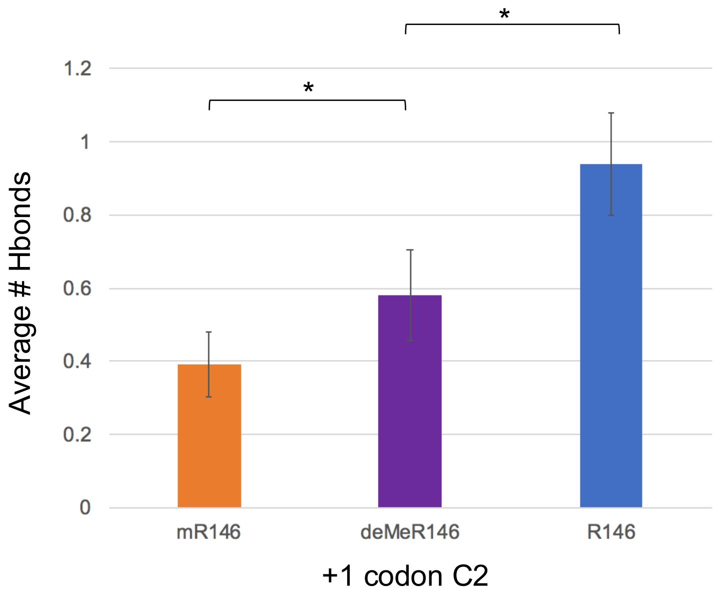

Figure S9. Demethylation of mR146.
The methyl group of mR146 was removed from the last frames of 20 MD trajectories (60-ns runs with N2 subsystem). After energy minimization, heating and equilibration steps, an additional 60 ns of MD was carried out and 20-60 ns of each run was analyzed (800 ns total). H-bond levels were graphed for interactions of the CAR interface with +1 codon C2. H-bond levels after demethylation (deMeR146, purple) were higher than the levels before demethylation (orange; paired t-test $p < 0.05$). H-bond levels of the demethylation runs were lower (two-sample t-test $p < 0.05$) than the levels detected with unmethylated R146 (blue; 20 X 60-ns runs). These results are consistent with our conclusion that R146 demethylation favors H-bonding with CAR.

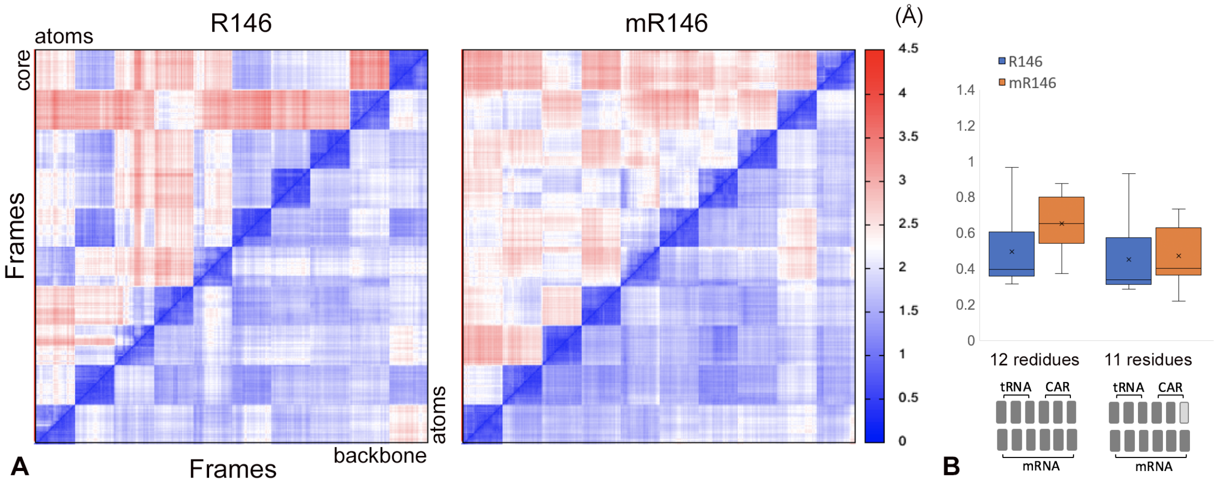

Figure S10. RMS2D analysis.
(A) RMS2D analysis was performed for 10 trajectories of 100 ns (excluding the first 20 ns) sampled at 1 frame/ns using backbone atoms (top right triangles) or "core" (base and guanidinium group) heavy atoms (bottom left triangles) of 12 residues (see Figure 5). (B) Similar to analysis of 60-ns trajectories (Figure 5), the differences across experiments comparing backbone and core RMSD were more pronounced for mR146 than R146 (but not significant in bootstrap tests).

1.2 Supplementary Tables

Table S1A. Unmethylated R146 Trajectories

R146 Trajectory Number	nt34-CAR Anchoring [1,2,3]	CAR Stacking[4]	CAR / mRNA Stacking[5]	A-site nt3 Interactions	+1 nt2 Orientation[6]	Other Comments
**1**	✓	CAR	R to +1 U3	X	out	
**2**	X	CA	A to +1 C2	nt34	up	R behind A
**3**	✓*	CAR	R to +1 U3	C1054	up	*nt34-C unstacks
**4**	✓	CAR	R to +1 U3	nt34	out	A-site nt1 pointed away from nt36
**5**	X	CAR	X	nt34	out	
**6**	✓	CA	A to +1 C2<br>R to +1 U3	nt34	out	
**7**	(X)	CAR	R to +1 C2	C1054	out	U3 pointed down
**8**	✓	CAR	X	C1054	up	R-U3 H-bonding
**9**	X	X	X	X	out	R behind A,<br>U3 pointed down
**10**	X	CAR	R to +1 U3	X	(up)	
**11**	(✓)	CAR	R to +1 U3	nt34	up	
**12**	(X)	X	A to +1 C2	nt34	out	A-site nt1 pointed away from nt36,<br>R behind A
**13**	✓	CAR	R to +1 U3	nt34	up	
**14**	X	CA/AR	X	nt34	up	C1054 perpendicular to nt34
**15**	X	X	A to +1 C2	nt34	up	R moves behind A
**16**	✓	CAR	R to +1 U3	nt34	up	
**17**	X	CA	A to +1 C2<br>R to +1 U3	nt34*	(out)	*nt34 rotated 90°
**18**	✓	CA	A to +1 C2<br>R to +1 U3	nt34, +1 G1	out	

**19**	X	CA	A to +1 C2	nt34	out	A-site nt1 pointed away from nt36
**20**	X	CAR	R to +1 U3, then R to +1 C2	nt34	up then out	
**21**	X	CAR*	R to +1 U3*	nt34	up	*R moves behind A then back to AR stacked
**22**	✓	CAR	R to +1 U3	nt34	up	
**23**	✓	CAR	X	nt34	up	A-site nt1 stacked with nt36, U3 pointed down
**24**	✓	CAR	X	nt34	up	U3 pointed down
**25**	X	CAR	R to +1 U3	C1054	out	A-site codon shifts forward 1nt and then back
**26**	✓	CAR	R to +1 U3	nt34	up	
**27**	✓	CAR/CA	R to +1 U3/ A to +1 C2	nt34	out	A-site nt1 pointed away from nt36, A-site nt2 Hbond to nt36
**28**	✓	CAR	R to +1 U3	nt34	up	
**29**	X	CAR	X	nt34	out	G1 rotated 90°, U3 pointed down
**30**	✓	CAR	A to +1 C2 R to +1 U3	nt34	out	

Table S1B. Methylated R146 Trajectories

**mR146 Trajectory Number**	**nt34-CAR Anchoring**[1,2,3]	**CAR Stacking**[4]	**CAR / mRNA Stacking**[5]	**A-site nt3 Interactions**	**+1 nt2 Orientation**[6]	**Other Comments**
**1**	✓	CAR to CA*	(A to +1 G1)	nt34	out	*R146 moves behind A1196
**2**	X	CA	(A to +1 C2 and R to +1 U3)	nt34	out	C1054 rotated 90°, R146 behind A1196
**3**	(✓)	CA	A to +1 C2,	(nt34)	out	R146 behind

			(R to +1 U3)			A1196
**4**	✓	CA(R)	X	nt34	down	U3 pointed down
**5**	X	CA to CAR*	X	C1054	up	nt34 rotated 90°, *R146 moves from behind A1196 to stacked
**6**	✓	CAR	R to +1 C2 to +1 U3	nt34	out	
**7**	✓	CA	A to +1 C2	(nt34)	out	R146 behind A1196
**8**	✓	CA	A to +1 C2, (R to +1 U3)	nt34	out	
**9**	X	(AR)	A to +1 G1, R to +1 C2	X	out	A site nt1 rotated 90°
**10**	X	(CR)	(A to +1 C2)	nt34	out	R146 behind A1196
**11**	✓	CA	(A to +1 C2), R to +1 U3	nt34	out	A-site codon shifted forward 1nt, nt34 unstacked from nt35
**12**	✓	CAR to CA*	(R to +1 C2 and +1 U3)	nt34	up	*R146 moves behind A1196
**13**	✓	CAR	R to +1 U3	X	up	nt34 rotated 90°
**14**	X	CAR	C to +1 G1, (R to U3)	nt34	up	
**15**	(X)	CA(R)	(A to +1 C2)	nt34	(up)	U3 rotated down
**16**	✓	CA(R)	(A to +1 C2), (R to +1 U3)	nt34	(up)	
**17**	✓ to X*	CA to X*	A to +1 G1	nt34	out	U3 rotated down, *R146 moves behind A1196
**18**	✓	CA	A to +1 C2	nt34/nt35	out	A-site codon shifted forward 1nt
**19**	✓	AR	R to +1 U3	X	(up)	A-site codon shifted forward 1nt
**20**	✓	CA	X	X	out	R146 behind A1196

**21**	✓	CAR	R to +1 C2	X	out	A-site nt3rotated 90°
**22**	✓	CA	A to +1 C2	nt35	out	U3 pointed down
**23**	(X)	(CR)	X	nt34	up	R146 behind A1196
**24**	X	CAR to CA*	X then A to +1 C2	nt34	out	G1 pointed toward A-site, *R146 moves behind A1196
**25**	✓	CA	X	nt34	up	R146 behind A1196
**26**	✓	CA	A to +1 C2 to +1 U3	X	up	R146 behind A119
**27**	✓	CA	A to +1 C2	(X)	out	
**28**	X	(CR)	(R to +1 C2)	nt34	out	R146 behind A1196
**29**	✓ to X	CAR to CA	R to +1 C2 to +1 U3, then X	nt34	out	
**30**	✓	CR	X	nt34	up	R146 behind A1196, G1 stacks with C1054 at the end

Notes:

[1] Entries in parenthesis show intermittent behaviors.

[2] Entries marked with * are discussed in the Other Comments column.

[3] nt34-CAR Anchoring describes if there was consistent stacking of tRNA nucleotide 34 with C1054 (✓).

[4] CAR Stacking shows reduced stacking with mR146 and the arginine is frequently located behind A1196.

[5] CAR/mRNA Stacking shows cases of CAR residues stacking with +1 codon nucleotides.

[6] +1 nt2 Orientation describes if the 2nd nucleotide of the +1 codon was oriented towards the CAR interface (up).

Table S2. Neighborhood 2 (N2) residues.

Chain	5JUP Numbering*	5JUP Restrained	Subsystem N2 Numbering	Subsystem N2 Restrained
A 18S rRNA	7-13	7, 12-13	1-7	1, 6-7
A 18S rRNA	558-583	558-562, 567-572, 582-583	8-33	8-12, 17-22, 32-33
A 18S rRNA	1000-1002	1000-1002	34-36	34-36
A 18S rRNA	1136-1152	1136-1144, 1151-1152	37-53	37-45, 52-53
A 18S rRNA	1176-1200	1176-1186, 1196, 1199-1200	54-78	54-64, 74, 77-78
A 18S rRNA	1207-1210	1207-1210	79-82	79-82
A 18S rRNA	1263-1291	1263-1267, 1285-1291	83-111	83-87, 105-111
A 18S rRNA	1299-1302	1299-1302	112-115	112-115
A 18S rRNA	1327-1329	1327-1329	116-118	116-118
A 18S rRNA	1419-1449	1419-1421, 1442-1449	119-149	119-121, 142-149
A 18S rRNA	1461-1465	1461-1465	150-154	150-154
A 18S rRNA	1621-1647	1621-1629, 1642-1647	155-181	155-163, 176-181
A 18S rRNA	1752-1772	1752-1753, 1766, 1769-1772	182-202	182-183, 196, 199-202
A 18S rRNA	1780-1783	1780-1783	203-206	203-206
AB S3	26-27	26-27	207-208	207-208
AB S3	102-120	102-114, 117-120	209-227	209-221, 224-227
AB S3	134-159	134-135, 153-159	228-253	228-229, 247-253
AB S3	170-187	170-171, 186-187	254-271	254-255, 270-271
AC S29	24-29	24-29	272-277	272-277
AC S29	39-45	39-45	278-284	278-284
B 25S rRNA	2253-2264	2253-2255, 2258-2264	285-296	285-287, 290-296
BC S30	2-17	2-17	297-312	297-312
CC S31	82-85	82-85	313-316	313-316
DC eEF2	574-588	574-576, 588	317-331	317-319, 331
DC eEF2	608-612	608-612	332-336	332-336
DC eEF2	631-635	631-635	337-341	337-341
DC eEF2	646-669	646-649, 667-669	342-365	342-345, 363-365
DC eEF2	686-713	686-689, 710-713	366-393	366-369, 390-393
DC eEF2	839-842	839-842	394-397	394-397
EC tRNA (IRES)	6895-6915	6895-6897, 6913-6915	398-418	398-400, 416-418
EC mRNA (IRES)	6946-6958	6946, 6958	419-431	419, 431
HB S10	59-62	59-62	432-435	432-435
NB S16	137-143	137-143	436-442	436-442
RB S20	67-82	67, 77-82	443-458	443, 453-458
UB S23	58-70	58-59, 68-70	459-471	459-460, 469-471
UB S23	115-118	115-118	472-475	472-475

**ZA S2**	83-98	83-86, 97-98	476-491	476-479, 490-491
**ZA S2**	118-120	118-120	492-494	492-494

*Same residue numbering for 5JUO, 5JUS, 5JUT, and 5JUU.

1.3 Supplementary Videos

Video S1. Trajectory with unmethylated R146 (separate file).
Representative trajectory sampled at 2 frames per ns (R146 trajectory number 16; Table S1A).

Video S2. Trajectory with methylated R146 (separate file).
Representative trajectory sampled at 2 frames per ns (mR146 trajectory number 1; Table S1B).

1.4 Supplementary Data File

Data File S1. Computer code for molecular dynamics analysis (separate file).
